# Potential of Neural Stem Cell-Based Therapy for Parkinson's Disease

**DOI:** 10.1155/2015/571475

**Published:** 2015-11-17

**Authors:** Chung-Hsing Chou, Hueng-Chuen Fan, Dueng-Yuan Hueng

**Affiliations:** ^1^Department of Neurology, Tri-Service General Hospital, National Defense Medical Center, Taipei City 11490, Taiwan; ^2^Tung's Taichung MetroHarbor Hospital, Wuchi, Taichung, Taiwan; ^3^Department of Neurological Surgery, Tri-Service General Hospital, National Defense Medical Center, Taipei City 11490, Taiwan

## Abstract

Neural stem cell (NSC) transplantation is an emerging strategy for restoring neuronal function in neurological disorders, such as Parkinson's disease (PD), which is characterized by a profound and selective loss of nigrostriatal dopaminergic (DA) neurons. Adult neurogenesis generates newborn neurons that can be observed at specialized niches where endothelial cells (ECs) play a significant role in regulating the behavior of NSCs, including self-renewal and differentiating into all neural lineage cells. In this minireview, we highlight the importance of establishing an appropriate microenvironment at the target site of NSC transplantation, where grafted cells integrate into the surroundings in order to enhance DA neurotransmission. Using a novel model of NSC-EC coculture, it is possible to combine ECs with NSCs, to generate such a neurovascular microenvironment. With appropriate NSCs selected, the composition of the transplant can be investigated through paracrine and juxtacrine signaling within the neurovascular unit (NVU). With target site cellular and acellular compartments of the microenvironment recognized, guided DA differentiation of NSCs can be achieved. As differentiated DA neurons integrate into the existing nigrostriatal DA pathway, the symptoms of PD can potentially be alleviated by reversing characteristic neurodegeneration.

## 1. Introduction

Parkinson's disease (PD) has traditionally been regarded as the most common neurodegenerative movement disorder, first reported by British physician James Parkinson in his 1817 essay as six cases of paralysis agitans [1]. Understanding of the disease was extended from clinical characteristics to a pathological diagnosis, when Frederic Lewy described microscopic particles in affected brains as early as 1912, later named “Lewy bodies” [1]. In the era of molecular biology, a diagnosis of PD is possible through the detection of mutations in specific genes that code for, for example, alpha-synuclein (SNCA) and parkin (PRKN), responsible for familial PD. Only about 10% of diagnosed patients carry identifiable pathological mutations though, and the majority of PD cases are sporadic [2]. Current pharmacologic treatments, including L-Dopa and monoamine oxidase-B inhibitors, as well as advanced surgical interventions such as deep brain stimulation (DBS), play no role in reversing the characteristic degeneration. As the prevalence of PD reaches 0.3% of the entire population in industrialized countries and 4% in those aged over 80 [2], a cure is urgently required to prevent suffering from nonmotor symptoms, including dementia, sleep disturbance, and autonomic dysfunction, as well as typical motor symptoms, such as asymmetrical bradykinesia, rigidity, postural instability, and resting tremors.

Replacement of lost dopaminergic (DA) neurons and accompanied tissues is a logical treatment for PD. Generally more than 50% of DA neurons have been lost before typical symptoms of PD develop [3]. Neural stem cell (NSC) transplantation is an attractive treatment option, as the cells have the capacity to self-renew and differentiate into all neural lineage cells, which may replace lost DA neurons and reverse the degenerative process of PD [4]. NSCs derived from iPSCs, or immortalized NSC lines, minimize the ethical conflicts using fetal ventral mesencephalic (VM) tissue for transplantation. Once standardized procedures for cell preparation and surgical techniques are established, a thorough evaluation of preclinical and clinical approaches will provide the evidence necessary for promoting cell-based therapies in PD. In this minireview, we highlight the significance of neurovascular unit (NVU) disruption in the progression of PD and introduce the advantages of NSC-based therapy.

## 2. NSCs and Neurogenesis in Adult Brains

Endogenous neurogenesis in the adult mammalian brain can be regarded as repair or replacement for neuronal loss [5]. Neuronal differentiation is largely restricted to two neurogenic niches where NSCs reside, the subependymal zone (SEZ) of the lateral ventricles and the subgranular zone (SGZ) of the dentate gyrus in the hippocampal formation [6, 7]. Only specific types of neurons, such as interneurons at the olfactory bulb (OB) and dentate granule cells in the hippocampus, are generated for replacing older neurons [8]. Several groups have shown that newly differentiated neurons can migrate to acutely injured areas, such as infarcts in ischemic stroke [9, 10]; however solid evidence is lacking for regenerated neurons replacing lost DA cells in PD.

The SEZ contains NSCs, astrocytes, immature precursors, neuroblasts, and ependymal and endothelial cells (ECs). The resident stem cell lineage in the SEZ consists of NSCs and the relatively quiescent neural progenitor cells (type B cells), which can give rise to rapidly dividing transit-amplifying cells (type C cells). Type C cells ultimately lead to migratory neuroblasts (type A cells), which enter the rostral migratory stream (RMS) [5]. A layer of ependymal cells lines the ventricle borders and is penetrated by the apical process of neural progenitor cells (NPCs), with a short single cilium at sites of adult neurogenesis. Beneath this layer, the bodies of type B cells are organized into chains of tunnels through which type A cells migrate. Proliferative clusters of type C cells reside along chains of type A cells, and within this layer runs an extensive network of blood vessels [12].

Neurogenesis is associated with the vasculature in neurogenic niches. NSCs are in close proximity to blood vessels, and so small molecules in the blood can regulate NSC regeneration [13, 14]. In the mouse brain the basal processes of NSCs frequently contact this vasculature, leading to a modified blood-brain barrier (BBB) at these sites of contact which lacks astrocytic end-feet and pericytic coverage [13]. NSCs lie closer to the SEZ vasculature, both in proximity and in frequency of direct contact. The close anatomical relationships of NSCs with ependymal cells and blood vessels and the direct contact with the CSF implicate signaling from each of these sources as potential regulators of stem cell behavior.

In addition to cellular components as a support for establishing and maintaining the microenvironment for NSCs to continue symmetrical divisions for self-renewal and asymmetric divisions for generating daughter cells, the composition and organization of the extracellular matrix (ECM) in the neurogenic niches are distinct from other regions of the adult brain [15]. The expression of receptors other than variations in the ECM is likely responsible for any contribution made by ECM signaling to NSC behaviors. It has been observed that fingerlike processes of basal lamina called fractones extend from blood vessels to contact each stem cell in the niche [16]. These structures contain laminin b1 and c1 chains, collagen IV, nidogen, and perlecan, with the laminin a1 chain present on blood vessels but not fractones [17]. Each stem cell receives different sources of laminin signals, and laminins composed of different laminin trimers may have different functions, which are essential for regulating the NSC behavior.

## 3. NVU Disruption in PD

The characteristic pathophysiology of PD includes death of DA neurons in the substantia nigra pars compacta (SNpc), degeneration of DA neurotransmission, and the presence of *α*-synuclein and protein inclusions in neuronal cells that are known as Lewy bodies [18]. In addition to gene mutations of specific proteins in familial or sporadic cases of PD, vascular insults to the brain as well as various environmental factors can induce PD-like symptoms, such as exposure to heavy metals, rotenone, or paraquat [19–21]. BBB disruption has been observed in each of these situations, based on clinical and* in vitro* evidence [22]. Importantly, *α*-synuclein deposition increases permeability of the BBB, suggesting the significance of *α*-synuclein in BBB disruption and PD development [23]. The implications of astrocytic modulation of the BBB have also been proposed in the development of PD [24]. In order to promote cell-based therapy in PD with the aim of regenerating DA neurons, it is essential to have a thorough understanding of the NVU, within which neurons exert their function of transmitting information through electrical and chemical signals.

The concept of the NVU has been extended over time to include a greater number of cell types, as the significance of interactions between these cells is revealed [25]. Initially, a triad of neurons, astrocytes, and vasculature was proposed as the NVU in 1996 [26]. The first recognized definition of the NVU was jointly proposed by the National Institutes of Health (NIH) and the National Institute of Neurological Disorders and Stroke (NINDS) in 2002 and comprised neurons, astrocytes, and ECs. According to this definition, both neural and endovascular elements are critical, especially for investigating cerebrovascular diseases, such as stroke.

Neurons are the signaling unit of neurotransmission; however their functional performance and maintenance rely upon complex interactions within a network of multiple cell types in the NVU. In addition to a physical association in the common microenvironment, these cells interact with each other through positive and negative feedback mechanisms, maintaining homeostasis. Astrocyte foot processes occupy 99% of the abluminal surface of brain capillaries, whereas neuronal endings may directly innervate either the capillary endothelium or the astrocyte foot processes investing the capillary endothelium [27]. Hemodynamic neurovascular coupling is essential for neuronal firing in the brain, as constant blood flow is required due to a lack of local energy reserve [28]. The ECs of the BBB can be distinguished from those in the periphery by an increased mitochondrial content, a lack of membrane fenestration, reduced pinocytic transport or transcellular flux, and extensive tight junctions which limit the influx of molecules between ECs known as paracellular flux [29–32]. Pericytes belong to a class of mural cells with contractile proteins and help maintain the structural integrity and function of blood vessels, being attached at irregular intervals to the abluminal membrane of the cerebral microvascular endothelium [33].

In contrast to the cellular compartments of the NVU, the acellular ECM is secreted by neighboring cells. These tissue-specific acellular matrices are primarily composed of proteins, polysaccharides, and enzymes and are the main component of the basement membrane (BM), which is an essential component of the BBB contributing to tissue and cell organization, stability, and differentiation. The neural ECM has a unique molecular composition, containing very small amounts of collagens, laminin-1, and fibronectin [34]. The matrix is mainly composed of a hyaluronic acid (HA) scaffold, which behaves as a large molecular sieving mesh that influences cell division and motility. Associated glycoproteins and proteoglycans regulate its molecular properties and spatial localization and are crucial in determining the major structural features of the neural ECM [35].

The BM of the BBB surrounds ECs and pericytes and presents a duplicature that separates the pericyte from the EC and the astrocyte end-feet. The formation and maintenance of the BM are ensured by ECs, pericytes, and astrocytes. The BM of the NVU is formed by tightly interwoven protein sheets of 20–200 nm thickness, created by the structural proteins collagen and elastin, as well as specialized proteins fibronectin and laminin, in addition to proteoglycans, organized in three layers: the lamina lucida, lamina densa, and lamina fibroreticularis [36]. The attachment of the BM to cells is primarily through laminins to sulfated glycolipids on the cell surface and transmembrane receptors [37]. The identification of dynamic regulation of tight junction proteins in human NSCs means that the cell-matrix interactions in the neurogenic niche, or potential target sites of NSC transplantation, will influence the therapeutic effects of NSCs in PD [38].

Regional heterogeneity has to be taken into account when NSCs are delivered into a specific target site for tissue regeneration, typically the putamen in PD brains. The importance of regional heterogeneity has been demonstrated in the central nervous system (CNS), with respect to cytoarchitectural organization in the NVU. Different interfaces exist for packaging neural tissue; for example, the BBB is distinct from the blood-cerebrospinal fluid (CSF) barrier, which is formed by choroid plexus epithelial cells and separates the blood from CSF. Within the neurogenic niche, a specialized NVU, ECs exert their influence over NSCs to regulate fate specification, differentiation, quiescence, and proliferation, through direct contact and paracrine signaling. In the SEZ, for example, a U-shaped gradient distribution of the soluble factor stromal cell-derived factor-1 (SDF-1), established by ependymal and endothelial cells, helps guide NSCs from the quiescent ependymal niche to the activated endothelial niche [39].

To promote NSC-based therapy in PD, the microenvironment of the target site of transplantation has to be thoroughly studied. Differentiation of delivered NSCs may simulate those in the endogenous neurogenic niches if a homeostasis can be established between the grafted cells and the surroundings. In theory, a series of descendants of NSCs will gradually replace the lost neurons through a sophisticated process of differentiation. The grafted NSCs are characterized by their capability of self-renewal through replication and differentiating into daughter cells by asymmetric division, in response to exogenous stimuli from the environment [40, 41]. As NSCs lose their stemness, the resulting NPCs cannot replicate indefinitely and instead have a tendency to differentiate into all neural lineage cells, such as neurons, astrocytes, and oligodendrocytes [42]. As they finally differentiate into migratory neuroblasts, the dividing cells are committed to the neuronal fate [43].

## 4. NSCs in PD

Adult neurogenesis may be affected in the progression of PD; for example, it has been observed that cell proliferation appears to be decreased in the SGZ and subventricular zone (SVZ) of PD patients. The numbers of proliferating cell nuclear antigen- (PCNA-) positive adult NSCs in the SVZ and nestin-positive precursor cells in the OB are decreased in the PD-affected postmortem brain, and a decrease in NSCs correlates with the progression of PD, while L-Dopa treatment appears to increase NSC numbers [44, 45]. In contrast, other groups have demonstrated no change in NSC proliferation in the PD-affected brain [46], suggesting that neurogenesis might be affected to various extents at different stages of PD. Strong evidence of a link between altered proliferation of NSCs and functional DA neurons remains insufficient.

PD can be regarded as a developmental disorder, in addition to a neurodegenerative disease, as more evidence suggests a relationship between deregulated neurogenesis and the onset and progression of the disease. It has been demonstrated that *α*-synuclein is necessary for the embryonic development of at least a subpopulation of DA neurons, shown using SNCA knockout mice as well as mice with a spontaneous deletion of the SNCA gene [47]. PD related SNCA mutations may lead to a reduction in the number of DA neurons in the developing brain long before the onset of DA neuronal degeneration. Reduced levels of *α*-synuclein contribute to an increased PD risk at the embryonic stage, whereas elevated *α*-synuclein levels are more commonly associated with PD in the degenerative stage. Disruption to the NVU results from increased permeability due to *α*-synuclein deposition during the development of PD, although altered levels of *α*-synuclein can be detrimental at different stages. Only an optimal concentration of *α*-synuclein is beneficial for DA neuron formation and maintenance, and this should be considered when generating DA neurons by NSC transplantation.

Research into embryonic development of the NVU is valuable when considering NSC-based therapy for PD, in order to replace lost DA neurons by neurovascular regeneration in the adult brain. In the developing brain the embryonic NSC microenvironment is influenced by blood vessels that begin to form as early as E9 [48, 49]. There are common signals, including VEGF, Notch, and Shh, which regulate both neurogenesis and angiogenesis [50]. The CNS vasculature develops through angioblastic invasion of the head region in early embryogenesis, and this vasculogenic process establishes the extracerebral vascular plexus that eventually covers the entire surface of the neural tube [51]. Further vascularization of the CNS, such as parenchymal capillaries, is exclusively achieved by angiogenesis from the perineural vascular complex (PNVP) [52]. Driven by metabolic demands of the expanding neuroectoderm, capillary sprouts invade from the extracerebral vascular plexus toward the periventricular zone [53]. The nascent brain vasculature is further stabilized by the formation of the ECM and the recruitment of mural cells, such as pericytes, and fine-tuned by cues from the microenvironment of neighboring cells [54]. BBB formation is not completely intrinsic to ECs in the CNS, and instead neural cells directly induce BBB characteristics in ECs from an early developmental stage [55]. The interactions between NSCs and ECs, through both direct physical contact and contact-independent signaling, begin with the recruitment of angioblasts and the formation of the PNVP in early embryogenesis [56]. Through this maturation process, all the components of the neurovascular network acquire their specific phenotypes and constitute a fully differentiated NVU.

As NVU disruption occurs in the progression of PD, DA neurons, associated astrocytes, and endothelium are all affected. To regenerate or restore the lost neural functions in the brain of PD with NSC-based therapy, the tissue regeneration should include not only DA cells, but also the other compositions of the neurovascular microenvironment. Additionally, an appropriate integration of DA neurons with the existing nigrostriatal DA system has to be established following DA neuronal differentiation of grafted NSCs.

## 5. Tissue and Cell Transplantation Therapy in PD

The symptoms of PD result from dysfunction or loss of DA neurons and associated neighboring tissue in the brain. Tissue regeneration and cell replacement is a potential approach for the neurodegenerative disorder, and since the late 1980s over 300–400 patients with PD worldwide have received transplants of human fetal VM tissue, rich in postmitotic DA neurons [57]. Two double-blind, placebo-controlled trials of VM transplants for PD patients, however, have shown variable efficacy and occurrence of side effects, including “off medication” or “graft induced dyskinesias” (GIDs) [58, 59]. It has also been observed that the PD pathologic process might propagate from host to grafted cells a decade after transplantation [60]. Long-term follow-up of one of these trials however showed consistent efficacy using both clinical and imaging outcome measures [61]. The inconsistent methods between the aforementioned trials, for example, immunosuppression and observation durations, may in part account for the disappointing results. In an attempt to overcome obstacles in the previous studies, new knowledge will be obtained from the TRANSEURO project, a multicenter European initiative on PD transplantation using fetal VM tissue (http://www.transeuro.org.uk). Issues still remain though, and heterogeneous compositions of the graft, difficulties in standardization of cell material, and ethical concerns are the fundamental limitations of transplantation therapy using fetal VM tissue.

As concept of the NVU replaces the traditional neurocentric view in neurologic disorders, such as PD and stroke, NSCs which preserve the ability to differentiate into all neural lineage cells and self-renew can be regarded as a potential graft for cellular transplantation. Neurons, astrocytes, and oligodendrocytes, which are differentiated from NSCs, together with ECs and pericytes, can constitute the functional NVU for tissue restoration in PD. Since neurons are integrated into the neurovascular network with other cellular and acellular compositions in the NVU, combined transplantation of NSCs with other types of cells or biomaterials may be more efficacious for tissue replacement. Besides the attempt to replace damaged tissues, it has been shown that cell-based therapies also promote endogenous vasculogenesis and neurogenesis in an animal model of ischemic stroke [62].

Technically DA neurons can be derived from embryonic stem cells (ESCs), mesenchymal stem cells (MSCs), umbilical cord blood hematopoietic stem cells (HSCs), and induced pluripotent stem cells (iPSCs) generated from adult somatic cells, as well as directly from NSCs [63]. In addition to identifying the most appropriate cell type, several factors including the long-term survival and phenotype stability of stem cell-derived neurons or glial cells in the graft following transplantation, the purity of populations of cells derived from NSCs, and safety issues related to the risk of tumorigenesis by grafted stem cells all need to be evaluated in greater depth [64]. Further investigations into the optimal therapeutic window, delivery route, cell dose, and patient selection are also required as a precondition for clinical translation of cell-based therapies [65].

To administer cell transplantation therapies, NSCs can be delivered through the needle into deep targets, such as putamen in the cases of PD. In contrast, the BBB seems to be an effective barrier preventing intravascular transplanted cells from crossing the vessel wall into brain tissue [70]. Typically, NSCs are injected transcranially into the brain parenchyma, close to the diseased tissues. It has been proposed that 100,000 surviving DA neurons per putamen are the minimum required for a successful outcome following intracranial transplantation in PD. Bilateral putamen grafts are favored more than unilateral transplantation, although there seems to be no consensus on the decision [71]. Transcranial transplantation of NSCs has been applied to experimental models of neurodegenerative diseases, such as PD [72], Huntington's disease [73], amyotrophic lateral sclerosis [74, 75], and Alzheimer's disease [76], in addition to vascular events, including ischemic stroke [77] and intracerebral hemorrhage [78].

Local factors within the microenvironment of transplanted NSCs affect the fate of the cells, as measured by survival, proliferation, differentiation, and neurogenesis [79]. Several groups have studied the modulation of grafted stem cells or DA cells with combined cellular transplantation in PD, although more extensive research is required into the topic (Table 1). To optimize survival and guide appropriate differentiation of grafted NSCs, ECs have been combined with NSCs for transplantation into stroke brains in animal models. Combined transplantation of ECs and neural stem and progenitor cells (NSPCs) increased survival and proliferation of ischemia-induced NSPCs and also accelerated neuronal differentiation [9]. A recent study demonstrates that cotransplantation of fetal DA neurons with mouse NSCs, genetically modified to overexpress human glial-derived neurotrophic factor (GDNF), mitigates motor symptoms in a rat model of PD [66]. It is reasonable to optimize the microenvironment surrounding the transplanted NSCs or DA neurons in order to support differentiation into DA cells and their survival* in vivo*.

## 6. Transplantation of Ensured Neurovascular Microenvironment Using NSCs

Several “clinical-grade” stem cell lines have been produced under Good Manufacturing Practice (GMP) conditions and according to established guidelines and safety regulations [80]. Recently a GMP clinical-grade NSC line manufactured by the technique of c-mycER^TAM^ transduction has been applied to the PISCES study (Pilot Investigation of Stem Cells in Stroke) [81]. This human NSC (hNSC) line was derived from the cerebral cortex of postmortem human fetuses at 10–12-week gestation [82]. It is genetically normal and stable over 50 population doublings. Genetic stability has been shown through G-banding karyotype analysis, Southern blot, and inverse PCR, in addition to phenotypic analyses by immunocytochemistry. These cells have been delivered intracranially in the treatment of patients with stable ischemic stroke (ClinicalTrials.gov Identifier: NCT01151124) [83]. Patients with PD will have the opportunity to receive cell-based therapy using these cells lines once DA neuronal differentiation can be guided appropriately.

The preclinical studies of NSC-based therapies, essential for improving neurobehavioral outcomes, should include investigations into a variety of factors, such as cell dose, timing of transplantation, and implantation sites [84]. Optimal experimental design for modeling the NVU should mimic as closely as possible the* in vivo* human brain where the grafted NSCs are to be delivered. With the technique of c-mycER^TAM^ transduction for NSCs derived from the whole ganglionic eminence and the ventral mesencephalon region of human fetuses, NSC lines have been induced and differentiated to neurons producing tyrosine hydroxylase (TH), a critical enzyme involved in dopamine synthesis [73, 85].

**Figure 1:**
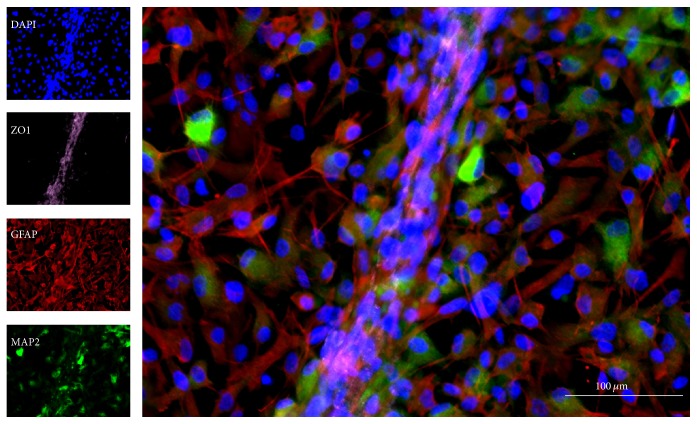
*In vitro* modeling of the neurovascular environment using a novel method of coculturing human NSCs with human cerebral microvascular ECs, showing distinctive cytoarchitecture [11]. The composition of the transplant, including NSCs as well as other cellular and acellular compartments, can be investigated through paracrine and juxtacrine signaling within the neurovascular unit (NVU). Microtubule associate protein-2 (MAP2) is a neuronal marker; glial fibrillary acid protein (GFAP) is an astrocyte marker; zonula occludens 1 (ZO1) indicates the presence of tight junction protein; diamidino-2-phenylindole (DAPI) serves as a nuclear counterstain.

Our novel model of coculturing hNSCs with human cerebral microvascular ECs (hCMECs) demonstrates a neurovascular environment, within which neuronal differentiation can be enhanced by neighboring ECs (Figure 1) [11]. The hNSC line has been shown to differentiate into cells positive for TH, and so these neurons could be guided to become DA neurons, replacing those lost* in vivo*. Using a novel method of modeling the NVU, unlike the Boyden chamber studies, we have shown the successful direct coculture of NSCs and hCMECs, demonstrating that direct interaction between the two cell types is possible [11, 38]. Intercellular communication is therefore possible through direct interaction of brain ECs with NSCs in vascular niches and may be essential for NSC self-renewal, proliferation, and differentiation. By understanding the interactions between NSCs and the surrounding compositions of the NVU, we can select NSCs with appropriate origins for the optimal transplant, ensuring the formation of the functional NVU and DA neuronal differentiation of NSCs. Once DA neurons are derived from transplanted NSCs and perform neurotransmission with integration into the existing nigrostriatal DA system, improvements in PD symptoms will be expected.

## 7. Perspectives on the NSC-Based Therapy for PD

NSCs are unique in that they can self-renew and differentiate into all neural lineage cells. They may therefore have the potential to replace lost neural tissue, such as DA neurons in PD. As NSCs are regulated by a specialized vasculature during adult neurogenesis, transplanted NSCs may be significantly affected by the neighboring vasculature and ECs, while they differentiate and integrate into the functional NVU. Elucidating interactions between NSCs and ECs should be an essential element of research and treatment plans for PD patients receiving transplants of NSCs.

Beyond cellular interactions, a number of technical parameters are likely to have an important role in cell-based therapy in PD, specifically issues of tissue procurement and preparation, such as the age and number of donor fetuses, graft dissection procedures, storage length and conditions, tissue dissociation before transplantation, or the use of ancillary neuroprotective strategies to increase graft survival (i.e., GDNF, lazaroids) [86, 87]. The fundamental concerns of cell-based therapies reside in the cells themselves, and major hurdles include devising methods for sustained clinical-grade production of immune-tolerable and genetically stable cells, prevention of tumorigenesis, improving graft survival accompanied by appropriate differentiation and integration of grafted or differentiated DA neurons into host circuits, and strategies to avoid untoward side effects like GID [88].

In this minireview we highlight the importance of establishing an appropriate neurovascular microenvironment into which NSCs, or any implants, are delivered in order to replace lost DA neurons. Using our novel model of NSC-EC coculture, it is possible to select appropriate NSCs and determine compositions of the transplant through complete investigation of the paracrine and juxtacrine signaling between elements of the NVU [11]. Once the cellular and acellular compartments of the graft and the microenvironment at the target sites are recognized, a guided differentiation of NSCs into DA neurons can be achieved. As these implanted or regenerated DA neurons are integrated into the existing nigrostriatal DA pathway, the symptoms of PD can potentially be alleviated by reversing characteristic neurodegeneration.

## Figures and Tables

**Table 1:** Modulation of stem cells or dopaminergic (DA) cells with combined cellular transplantation in PD.

Type of transplanted cells		Animal model	Significance	Reference
Mouse fetal DA neurons	Mouse mesencephalic NSCs overexpressing human glial-derived neurotrophic factor (GDNF-mNSCs)	6-OHDA rat	Apomorphine-induced rotation was reduced by cotransplantation of fetal DA neurons with mNSCs genetically modified to overexpress GDNF, which supports differentiation into DA cells and their survival	[66]

Human embryonic NSC	Macaque autologous Schwann cells (SCs)	6-OHDA macaque	Gomez-Mancilla dyskinesia score in the group of cotransplantation with SCs and NSCs was significantly lower than the control group. SCs harvested from the autologous peripheral nerves can avoid rejection	[67]

Human umbilical cord-derived MSCs	Human dermal fibroblasts	MPTP rat	Fibroblasts may be common cell contaminants affecting purity of MSC preparations and clinical outcome in stem cell therapy protocols	[68]

Rat embryonic DA neurons	Rat Schwann cells (SCs) overexpressing basic fibroblast growth factor (FGF-2)	6-OHDA rat	Cotransplantation of DA neurons and FGF-2 overexpressing SCs differentially affects survival and reinnervation. Behavioral recovery underlines the necessity of direct contact between FGF-2 and DA neurons	[69]
